# On the temporal dynamics of reward utilization in dual-task situations

**DOI:** 10.3758/s13414-025-03058-x

**Published:** 2025-04-07

**Authors:** Leif E. Langsdorf, Torsten Schubert

**Affiliations:** Institute for Psychology, Martin Luther University Halle-Wittenberg, Halle, Germany

**Keywords:** Dual-tasking, Reward utilization, Motivation, Temporal preparation

## Abstract

In dual-task (DT) situations, performance typically deteriorates compared with single-tasking situations. These decrements can be explained by the serial scheduling of response selection stages constituting a central bottleneck as with decreasing stimulus-onset asynchrony (SOA) the reaction time for the second task (Task 2; RT2) increases. Prior studies indicated that the reaction time for the first task (Task 1; RT1) and RT2 are improved in reward compared with no-reward conditions for a block-wise reward prospect, which reflects reward-related optimization in DT processing. However, it remains unclear whether participants can flexibly utilize reward information in a trial-by-trial manner to achieve reward-related improvements. Additionally, it is unclear whether a potential reward-related optimization reflects optimized task preparation only or whether the prospect of reward can evoke an *additional* task optimization mechanism that extends beyond preparation-related processing improvements. For Experiment 1, we combined a trial-wise reward prospect for participants' Task 1 performance, which was signaled by a cue before Task 1 onset, with block-wise presented cue–target intervals (CTI) of either 200 ms *or* 700 ms, resulting in precise temporal predictability of Task 1 onset by participants. First, we observed a reduced RT1 in the reward compared with the no-reward condition. Furthermore, the reward effects increased on RT2 for short compared with long SOAs, reflecting effect propagation at short SOA from Task 1 onto Task 2. Second, RTs decreased with increasing CTI, while reward effects increased with increasing CTI. Consequently, preparation-related processing improvements of DT performance were *additionally* improved by reward utilization. For Experiment 2, temporal predictability of Task 1 onset was reduced compared with Experiment 1 by presenting CTIs randomized within blocks, which allowed replicating the result pattern of Experiment 1. Across both experiments, the results indicate that participants can flexibly utilize reward information in a trial-by-trial manner and that reward utilization *additionally* improves preparation-related processing improvements for DT conditions with predictable and less predictable Task 1 onset.

## Introduction

The execution of two tasks in close temporal succession is difficult for humans. In such dual-task (DT) situations, participants’ performance usually declines compared with when the same tasks are performed apart. The cognitive processing architecture has long been investigated using DT paradigms such as the psychological refractory period (PRP) paradigm. In these PRP situations, participants execute two temporally overlapping choice reaction time (RT) tasks separated by a varying time interval between them (i.e., the stimulus onset asynchrony [SOA]; Pashler, 1994). This task situation usually results in declined performance compared with single-task situations, which are referred to as DT costs. Specifically, these DT costs relate to a performance pattern in which the response times of the second task (Task 2) are increased with decreasing SOA between both tasks. These costs can be explained with the central bottleneck model, which assumes the serial processing of the central response selection stages, while peripheral stages (i.e., perceptual and motor stages) are assumed to be processed in parallel. In contrast, it is assumed that for the short SOA condition, the response selection and motor stage of Task 2 are not processed until the response selection of the first task (Task 1) has been processed. This leads to a delay of Task 2 processing and explains why the reaction time to Task 2 (RT2) is increased at short SOA and decreases with increasing the SOA between tasks. At the same time, the reaction time to Task 1 (RT1) is assumed to be unaffected by the length of the SOA between Task 1 and Task 2.

Despite the debate about whether the processing limitations during DT have strategic or structural reasons, several factors have been linked to the modulation of DT processing, such as training (Ruthruff et al., 2006; Schubert & Strobach, 2018; Strobach et al., 2014), age (Hein & Schubert, 2004; Strobach et al., 2012), and different combinations of input and output modalities (Hazeltine et al., 2006; Stelzel et al., 2006). A further current question in this line of research is how the prospect of reward affects cognitive processing during DT (Fischer et al., 2018; Han & Marois, 2013; Rieger et al., 2021).

Previous investigations revealed that the prospect of reward for either Task 1 or Task 2 performance in a PRP DT situation leads to substantial DT improvements (i.e., reduced RT1 and RT2 in the reward compared with the no-reward conditions; Langsdorf et al., 2022, 2025). In these studies, the prospect of reward was manipulated block-wise. In detail, before each reward block, participants were instructed that they could receive a reward for fast and accurate Task 1 performance while minding low error rates on Task 2 performance. Importantly, in these reward blocks, each trial was reward-relevant reflecting a constant prospect of reward for the participants. Thus participants could apply a constant strategy of reward-induced preparation for an entire reward block to obtain a reward. In contrast, it remains unclear whether participants can utilize randomly changing trial-wise reward information and whether the prospect of reward can rapidly build up to improve DT performance (Fischer et al., 2018; Rieger et al., 2021; Yildiz et al., 2013). A further central yet open aspect is whether the utilization of reward information is affected by the length of the preparatory interval, or whether this is not the case. We addressed these open questions, investigating as a first aim of the present study whether participants can flexibly utilize the prospect of reward from trial to trial, as indicated by a cue. For the second and more central aim, we focused on the investigation of the temporal dynamics of reward utilization for behavioral adjustments in DT situations.

By now, there is consensus in the field that cueing the prospect of reward before a trial improves preparatory processes leading to enhanced task performance as reflected by reduced RTs (Chiew & Braver, 2016). In addition, physiological evidence indicates that the prospect of reward can improve motor preparation as well as modulate pupil dilation reflecting preparatory effort (Bundt et al., 2016; Chiew & Braver, 2013). Further evidence stems from event-related potentials (ERPs) with high temporal resolution indicating an earlier onset of task-related preparation processes in a reward compared with a no-reward condition (Schevernels et al., 2014). This questions the idea that reward effects on task performance go beyond pure preparation-related performance improvements (e.g., Rieger et al., 2021; Zedelius et al., 2012). In sum, accumulating evidence indicates a close link between reward-related and preparation-related processing improvements. However, further investigations are still required that investigate in more detail the temporal dynamics of reward utilization.

Related to that, Kleinsorge (2001), provided evidence for the assumption that the length of preparatory intervals affects the reward utilization of participants for processing improvements. In this study, participants were asked to discriminate letters, while at varying intervals before letter presentation, a cue was presented, which signaled whether or not to exhibit additional mental effort. If participants would respond with increased response speed, while committing few errors, they would receive a monetary reward. These so-called effort trials amounted to 20% of trials while the remaining 80% of trials were so-called standard trials. The author reported that with a preparatory interval between 600 and 900 ms, before letter onset, reward utilization for effort trials peaked, while the length of the preparatory interval did not improve task performance for the standard trials. Taken together, the results provide evidence for the assumption that the prospect of reward can be more effectively utilized with an increasing preparatory interval (see also Chiew & Braver, 2016; Falkenstein et al., 2003). Similarly, Chiew and Braver (2016) suggested that increased preparatory intervals enable improved encoding and processing of the reward information for processing improvements. However, it remains an open issue whether similar processing improvements would emerge in DT situations, because these task conditions are more demanding compared with single-task conditions. Furthermore, it remains an open issue whether participants could improve their task performance in a larger proportion of trials, because in previous studies effort trials were reduced in number compared with standard trials (Falkenstein et al., 2003; Kleinsorge, 2001; Steinborn et al., 2017).

A suitable methodological approach, for studying the temporal dynamics of reward utilization can be adapted from studies investigating temporal preparation in sensory-motor RT tasks (see also Falkenstein et al., 2003; Kleinsorge, 2001). In these experiments, preparatory intervals with varying lengths are presented before the target onset. The application of varying preparatory intervals enables the investigation of how temporal preparation impacts task performance, leading to a modulation of RTs, reflecting the temporal preparation effect (Niemi & Näätänen, 1981; Steinborn et al., 2017; Teichner, 1954).

In an investigation by Fischer et al. (2007), different levels of preparation for target processing were applied to test the effect of temporal preparation on the size of the subliminal priming (SP) effect. In SP tasks a target requiring a response is preceded by a subliminal prime, which is either associated with the same motor response (congruent), or with the opposite motor response (incongruent), as the target. Usually, this results in improved RTs in the congruent compared with the incongruent condition, while the SP effect is the difference in RTs of incongruent and congruent trials.

Fischer et al. (2007) presented either an accessory tone stimulus or no tone (serving as the control condition) followed at varying intervals by the presentation of a prime–target pair for which participants were asked to discriminate the pointing direction of the target arrow. For Experiment 1, a randomized presentation of tone–target intervals was applied inducing temporal uncertainty in the participants as they could not establish a precise temporal expectation of target onset. In contrast, for Experiment 2, a blocked presentation of tone–target intervals was chosen, resulting in temporal certainty of target onset. The application of a randomized and blocked presentation of tone–target intervals enabled Fischer et al. (2007) to explore how temporal expectation and temporal preparation jointly affect the SP effect.

The results across both experiments indicated improved task performance in the congruent compared with the incongruent condition. Furthermore, in comparison to the no-tone condition, the size of the SP effect in the tone condition increased with increasing preparation time. These results indicate that the tone stimulus was utilized as a temporal reference for response preparation during the preparatory intervals. The authors inferred that for longer preparatory intervals in contrast to shorter preparatory intervals, an enhanced pre-motoric response activation for stimulus processing can have occurred (Niemi & Näätänen, 1981; Steinborn et al., 2017). Taken together Fischer et al. (2007) provided evidence for the assumption that a temporal reference in combination with temporal preparation can improve the pre-motoric response activation leading to enhanced task performance in an SP task.

The assumption of a pre-motoric locus of the temporal preparation effect was further supported by the findings of studies applying electrophysiological methods. In particular, MÜller‐Gethmann et al. (2003) measured motor-related ERPs and reported an effect of temporal preparation on the stimulus-locked lateralized readiness potential (sLRP), which is considered to reflect processes before motor execution. In contrast, no effects of temporal preparation on the response-locked LRP (LRPr) were obtained which is considered to reflect processes related to motor execution. This pattern of results indicates an effect of temporal preparation on early pre-motoric processes and is in line with the assumptions and findings of Fischer et al. (2007; Bausenhart et al., 2006; Hackley & Valle-Inclán, 1998; Jepma et al., 2009).

A further specification of the pre-motoric locus of the temporal preparation effect stems from the application of an accumulation model of human information processing by Grice (1968). The model describes the process of information accumulation with three parameters: 1) the onset of information accumulation, 2) the rate of information accumulation, and 3) the internal decision criterion. In an empirical investigation, Seibold et al. (2011) provided evidence for the assumption that temporal preparation improved the onset of sensory information accumulation in a sensory-motor RT task. The authors concluded that an earlier onset of the sensory information accumulation for task conditions of high temporal preparation compared with low temporal preparation led to a faster reaching of the decision criterion. Taken together, converging evidence of studies applying different methodologies for investigating the temporal preparation effect indicates that temporal preparation improves pre-motoric processes as early as the onset of sensory information accumulation (Bausenhart et al., 2010; Rolke & Hofmann, 2007).

**Fig. 1:**
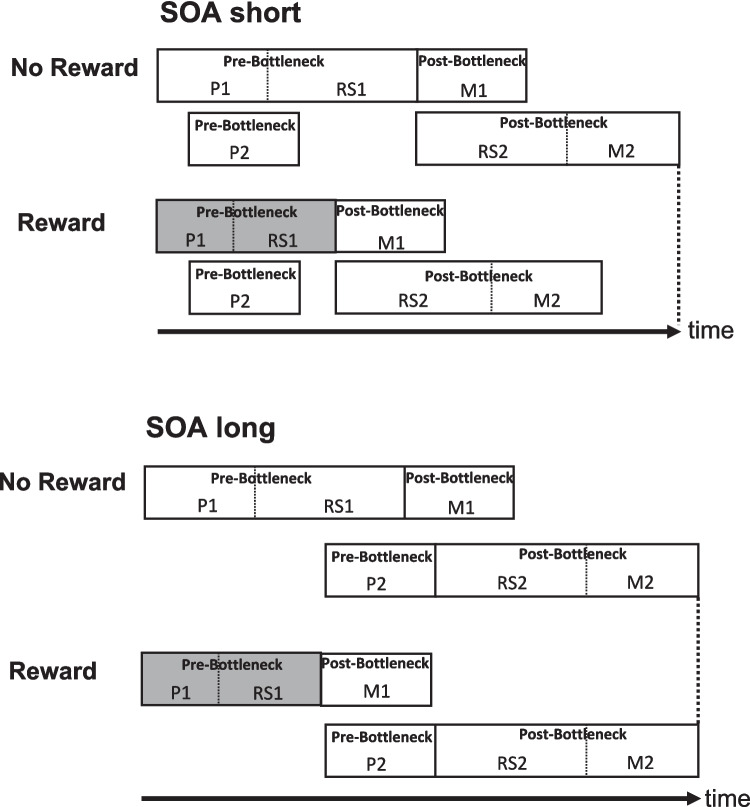
Depiction of the case when reward reduces the processing time of the pre- and/or bottleneck stages of Task 1, in the reward compared with the no-reward condition. The gray-shaded areas of Task 1 indicate that reward shortens the pre- and/or bottleneck stages of Task 1. P1 = perception stage of Task 1; Bottleneck stage of Task 1 comprises: RS1 = response selection of Task 1. Post-Bottleneck stage of Task 1 comprises M1 = Motor stage of Task 1; Pre-Bottleneck stage of Task 2 comprises: P2 = perception stage of Task 2; Bottleneck stage of Task 2 comprises: RS2 = response selection stage of Task 2; Post-Bottleneck stage of Task 2 comprises: M2 = motor stage of Task 2; SOA = stimulus-onset asynchrony

The present study had two research aims: first, we investigated whether participants can flexibly utilize a trial-wise reward prospect for their Task 1 performance. In the corresponding paradigm, a cue indicating whether or not the current trial is reward-relevant is presented shortly before Task 1. Importantly, cue identity varies randomly from trial to trial and participants need to adapt their reward utilization as well between different trials. If participants flexibly utilize the trial-wise reward information then the resulting RT pattern should resemble the RT pattern of previous studies in which the prospect of reward was applied for participants' Task 1 performance but was implemented block-wise. For such a case, we predict an improved RT1 in the reward compared with the no-reward condition, as well as larger reward effects on RT2 at short compared with long SOA. According to the effect propagation logic, such an RT pattern emerges, if the processing stages of Task 1 before or/at the bottleneck are shortened leading to an improved RT1 (see Fig. 1). As a result, the change in the processing duration will be propagated via the bottleneck mechanism onto the Task 2 processing chain, improving also RT2 (Johnston & Pashler, 1990; Langsdorf et al., 2022; Schubert, 1999). Consequently, the effects on RT2 should be increased at short compared with long SOA, as the lack of the bottleneck mechanism at long SOA prevents the propagation of RT changes of the processing stages before or/at the bottleneck of Task 1 onto Task 2. Taken together, this should result in a main effect of reward on RT1 and an overadditve interaction of reward and SOA on RT2, with larger reward effects at short compared with long SOA. The emergence of such an RT1 and RT2 pattern would suggest, that participants were able to flexibly utilize reward information for processing improvements from trial-to-trial and this would represent a good starting point for elucidating the temporal dynamics of reward utilization.

For the second and more central research aim, we investigated the temporal dynamics of reward utilization. To that end, the size of the reward effect for participants' Task 1 performance was compared between different durations of the cue–tasrget interval (CTI), which was manipulated between blocks (i.e., of either 200 ms or 700 ms). In *each* DT trial, a cue was presented, signaling to the participants whether or not the upcoming DT trial would be reward-relevant, leading to a 50/50 proportion of reward and no-reward trials. As a result, this enabled participants to utilize the cue as a temporal reference for response preparation in the reward and no-reward conditions. However, in the reward condition, the cue could additionally (i.e., compared with the temporal preparation-related effects) improve response processing by stimulating reward-related performance improvements.

Concerning the issue of the temporal dynamics of reward utilization several predictions can be derived. Under the assumption, that participants can flexibly utilize reward information between trials, it is conceivable that the utilization of reward information improves with an increasing preparatory interval, following the results of Kleinsorge (2001) from single-task situations (and others: Chiew & Braver, 2016; Falkenstein et al., 2003). If that was the case, the utilization of reward information should *further* increase with an increasing length of the preparatory interval, resulting in larger reward effects for the long CTI compared with the short CTI condition on RTs. For the current case, in each trial, a cue was presented that participants could utilize as a temporal reference for response preparation. If, in addition, the cue signaled the prospect of reward in the long CTI condition, DT performance in the reward compared with the no-reward condition should improve to a larger extent compared with the short CTI condition.^1^ This would result in larger reward effects in the long compared with the short CTI condition on RTs, as reflected by an overadditive interaction of CTI and reward on RTs.

Now let us consider the scenario in which reward utilization improves with increasing CTI, while temporal preparation is also optimized as CTI increases. Improved temporal preparation would manifest as shorter RTs in the long CTI compared with the short CTI condition by itself. In this context, a combined effect of both (i.e., of temporal preparation and reward) would be characterized by an overadditive interaction between CTI and reward on RTs, alongside a main effect of CTI on RTs. Such a pattern would indicate that reward utilization enhances task performance beyond temporal preparation alone. In other words, such a pattern would mean that optimally prepared RTs would further benefit from improved reward utilization in the long CTI condition compared with the short CTI condition. Such findings would challenge the assumption that reward-related processing improvements are solely attributable to preparation-related optimization processes (e.g., Rieger et al., 2021; Zedelius et al., 2012). Instead, such findings would suggest the presence of an additional optimization process, potentially involving enhanced information processing.

As a further alternative, we should also consider that participants may lack the ability to flexibly utilize reward information for processing improvements on a trial-by-trial basis at all. Such an assumption could also be derived as previous DT studies investigating reward processing only applied variants of block-wise reward manipulations (Fischer et al., 2018; Langsdorf et al., 2022, 2025; Rieger et al., 2021; Yildiz et al., 2013). If this was the case, participants' reward utilization would likely remain ineffective regardless of the CTI condition. As a result, no significant differences would be expected between the reward and no-reward conditions for either CTI condition on RTs.

To investigate the temporal dynamics of reward utilization in more detail, we *additionally* focused on the analysis of the RT distribution. In particular, a Vincentized cumulative RT distribution analysis enables one to observe the effects of a reward and the preparation manipulation on the percentiles of the RT distribution. Thus, providing a more fine-grained tool for the interpretation of the potential effects on the mean RTs (Ratcliff, 1979; Schubert et al., 2002; Steinborn et al., 2017). Consequently, we analyzed whether the effects on the RT mean are driven by the speed-up of RTs at the different tails of the distribution. We hypothesized that during optimally prepared trials with shorter RTs cognitive processes are executed efficiently, thus no further improvements are expected by the prospect of reward, irrespective of the CTI condition (De Jong, 2000). In contrast, during trials with longer RTs, i.e. the right tail of the distribution, the prospect of reward might further optimize the cognitive processing chain. This is conceivable since earlier studies reported an effect of mental effort on the right tails of the RT distribution, assuming improved attentional mobilization behind this effect (Steinborn et al., 2017; Strayer et al., 2024). Concerning the current investigation, the effect of reward prospect on the right tail could be more pronounced in the long CTI condition compared with the short CTI condition, as the effect of reward prospect might build up over time, leading to increased reward effects. This effect pattern would indicate that the utilization of reward information is improving with increasing CTI.

## Experiment 1

In Experiment 1, we investigated two research aims, first, we tested whether participants could flexibly utilize a trial-wise reward prospect for their Task 1 performance. Second, we were interested in the temporal dynamics of reward utilization. To that end, we administered a cue in the reward and the no-reward condition before Task 1 presentation and manipulated the CTI block-wise either for 200 ms or 700 ms. The task situation comprised of an auditory-visual DT which was separated by one of three SOAs (100 ms, 300 ms, or 900 ms). Furthermore, participants were instructed that they could earn a monetary reward if their response to Task 1 was fast and accurate while maintaining a low error rate in Task 2.

### Materials and methods

#### Participants

Twenty-five healthy participants (21 women; *M*_age_ = 22 years) were invited to take part in the experiment after obtaining written informed consent and were debriefed after the session. We used the *superpower* R package (Lakens & Caldwell, 2021) a Monte Carlo simulation-based tool for conducting power analyses^2^ for multifactorial within-designs. To estimate the required sample size for the interaction effect of the factors reward and CTI on RTs (i.e., a larger reward effect in the long CTI condition compared with the short CTI condition). Absolute RTs for each cell, standard deviations, and correlation coefficients among within-subject factors were estimated based on a pilot study and a previous DT investigation (Langsdorf et al., 2022). Setting *α* = 0.05, the simulation analysis yielded a sample size of *N* = 25 for detecting an interaction effect with the power of 90%. The experimental protocol conformed to the declaration of Helsinki. All participants were right-handed, German native speakers, and had normal or corrected-to-normal vision. Furthermore, participants could choose between 4 euro or course credit as a general payment, which was added to the performance-dependent amount of monetary reward (see below).

#### Apparatus and stimuli

Participants performed a PRP DT consisting of an auditory and a visual choice RT task. Stimuli for the auditory task comprised three sine-wave tones with a frequency of 250, 500, or 1000 Hz presented for 200 ms via headphones. Participants responded to the low-, middle-, and high-pitched tones by pressing the ‘Y’, ‘X’, and ‘C’ keys of a QWERTZ keyboard with the ring, middle, and index fingers of their left hand, respectively. For the visual task, one of three digits (1, 5, or 9) was presented centrally on a computer screen with a visual angle of 1.07° × 1.07° at a viewing distance of 80 cm. Visual stimuli appeared for 200 ms, and participants responded to the digits in ascending order by pressing the keys ‘M’, ‘,’, and ‘.’ of a QWERTZ keyboard with the index, middle, and ring finger of their right hand, respectively. Participants were instructed to first respond to the auditory and then to the visual task. Each trial started with the presentation of a fixation cross at the center of the screen for 950 ms followed by a white or blue ring with a visual angle of 2.15° × 2.15° (indicating either a reward or no-reward trial) for 200 ms followed by a blank interval of either 0 ms or 500 ms, depending on the CTI condition of 200 ms or 700 ms. Subsequently, the auditory stimulus was presented for 200 ms, followed by the visual stimulus for 200 ms, separated by an SOA of either 100 ms, 300 ms, or 900 ms. After a response to both target stimuli or a maximal response duration of 3,000 ms, an intertrial interval of 500 ms followed before the start of the next trial. Participants received the feedback “Falsch” (German for *wrong*) for 500 ms if either one or two of their responses were erroneous. If their response to either target exceeded the maximal response duration, the feedback “Zu langsam” (German for *too slow*) was presented for 500 ms. If participants responded first to the digit task, the feedback “Reihenfolge beachten” (German for *mind response order*) was presented for 500 ms.

#### Design and procedure

We applied a three-factorial within-subjects design, with reward, CTI, and SOA as independent variables. Each block consisted of 54 trials resulting from the combination of two reward levels (reward or no reward), three SOAs (100 ms, 300 ms, 900 ms), three auditory stimuli (250 Hz, 500 Hz, 1000 Hz), and three visual stimuli (1, 5, 9). In total, six DT blocks were presented comprising three DT blocks with a CTI of 200 ms and three DT blocks with a CTI of 700 ms, resulting in 324 experimental trials.

The procedure was as follows: The experiment started with a single-task practice phase in which participants performed 12 single-task trials for each component task (auditory and visual). The timing of these single-task trials was identical to DT trials with the exception that only one target stimulus was presented and only one response was required. These single-task trials were followed by two blocks of 54 trials of DT practice one block for each CTI condition. At the start of the DT practice, participants were instructed to respond to Task 1 as soon as it was presented (Ulrich & Miller, 2008).

After that, participants entered the reward phase of the experiment. For that purpose, they were instructed that their Task 1 performance was rewarded with 72 euro cents per block. They would receive a reward if their response to Task 1 was fast and accurate, while their Task 2 performance was not rewarded (to mind low error rates on Task 2). The color of the cue signaled either a reward or no-reward trial presented either in white or blue (counterbalanced across participants). The information about the cue–reward mapping and the reward scheme was again presented before each block. Furthermore, the experiment only proceeded after the participants had verbally reported the cue–reward mapping and the reward scheme (Chiew & Braver, 2016). For the first reward block, we set a pre-defined threshold of 850 ms for Task 1 performance as well as 89% accuracy, based on previous studies (Langsdorf et al., 2022; and pilot studies). Subsequently, participants' thresholds for earning a reward were continuously calculated based on their mean RT1 performance and their mean error rates in reward blocks.

If in the first reward block either participants' mean RT1 or their mean error rates met the pre-defined threshold, they received 72 euro cents. If none of their performance measures were below the pre-defined thresholds, they received no reward. Thereafter, the threshold RT1 was updated by averaging the pre-defined threshold (850 ms) and the mean RT1 of the previous reward block. Similarly, the mean error rate was updated. Subsequently, only the performance measures from the reward blocks were used to compute individual thresholds for obtaining a reward. After each block, participants received feedback about their mean RT1 and percentage of correct trials, and whether they earned a reward (and how much reward they had earned so far). Finally, participants were naïve about the threshold computations for obtaining a reward.

#### Statistical analysis

Mean RTs and error rates were analyzed separately for RT1 and RT2 using an analysis of variance (ANOVA) with the within-subjects factors reward, SOA, and CTI. A significance threshold of 5% was used for all analyses. The *p* values of the ANOVAs were adjusted according to the Greenhouse–Geisser correction when necessary (Huynh, 1978). For the RT analyses, trials with at least one erroneous response (*M* = 7.7%) and outliers that deviated more than ± 2.5 standard deviations (*SD*s) for each participant and factor combination (*M* = 4.9%) were excluded from the data set. Furthermore, trials were excluded that met the criterion of response grouping (RT2 − RT1 + SOA) < 200 (Ulrich & Miller, 2008). All analyses and visualizations were conducted in R and ggplot2 relying on the tidyverse dialect (R Core Team, 2021; Wickham, 2011; Wickham et al., 2019).

### Results

#### Task 1

**Fig. 2:**
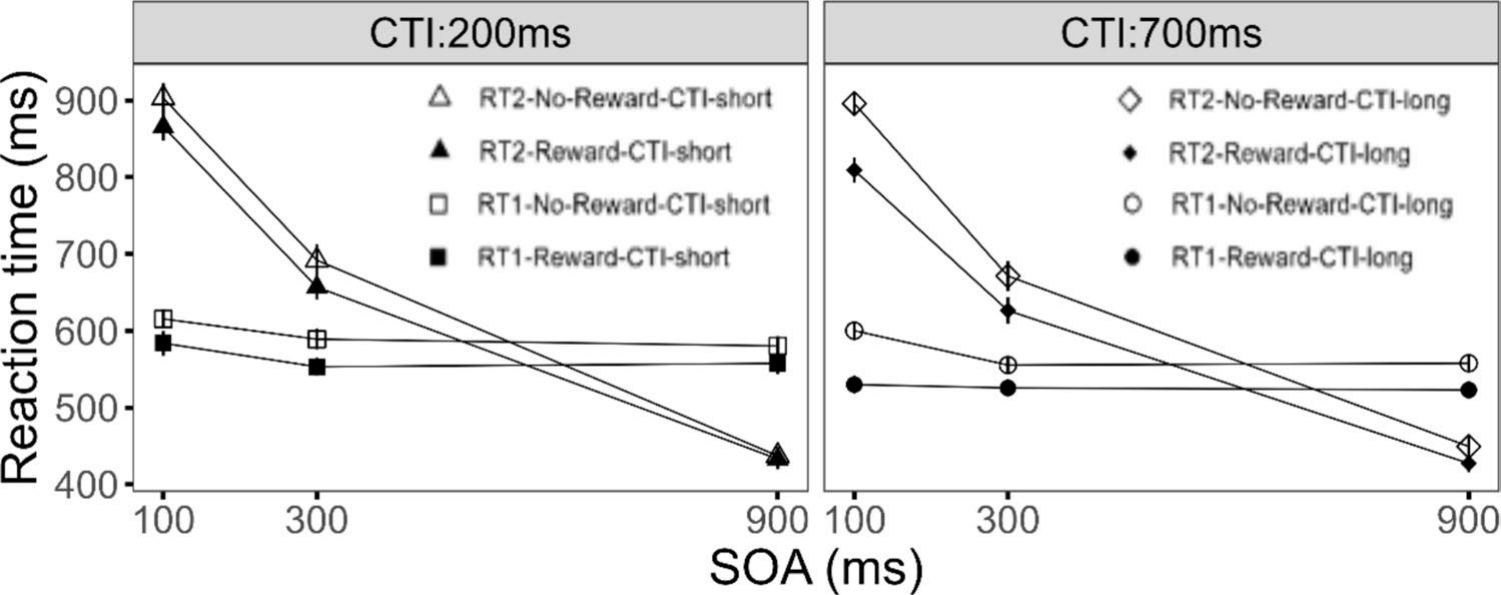
Mean RTs for Task 1 and Task 2 as a function of reward, stimulus-onset asynchrony (SOA), and cue–target interval (CTI) for Experiment 1. Error bars represent the standard error of the mean

We first tested for the effects of reward on Task 1 performance. We obtained a significant main effect of the factor reward, *F*(1, 24) = 18.26, *p* < 0.001, η_G_^2^ = 0.053. Participants’ RT1 was reduced in the reward (*M* = 546 ms) compared with the no-reward (*M* = 583 ms; see Fig. 2) condition. The interaction of the factors reward and SOA reached significance, *F*(2, 48) = 3.90, *p* < 0.027, η_G_^2^ = 0.004, with marginally increased reward effects at SOA 100 (*M* = 51 ms) compared with SOA 300 (*M* = 33 ms), *t*(24) = 2.01, *p* = 0.056, and larger reward effects compared with SOA 900 (*M* = 29 ms), *t*(24) = 2.57, *p* < 0.017. Taken together these reward effects indicate that participants were utilizing the reward information to improve their DT performance. Furthermore, we obtained a significant main effect of the factor SOA, *F*(2, 48) = 9.84, *p* < 0.001, η_G_^2^ = 0.026, on RT1, with an increasing RT1 for shorter SOAs. Such effects of SOA on Task 1 are often explained by participants’ tendency for response grouping (Strobach et al., 2015; Ulrich & Miller, 2008). In addition, we obtained a significant main effect of the factor CTI, *F*(1, 24) = 21.76, *p* < 0.001, η_G_^2^ = 0.038, with a reduced RT1 in the long CTI (*M* = 548 ms) compared with the short CTI (*M* = 577 ms) condition, indicating the typical preparation effect on RT1 performance (Niemi & Näätänen, 1981).

Most importantly for the issue of the temporal dynamics of reward utilization, we obtained a significant overadditive interaction of the factors reward and CTI on RT1, *F*(1, 24) = 4.76, *p* < 0.039, η_G_^2^ = 0.002. In particular, the reward effect in the long CTI condition (*M* = 45 ms) was increased compared with the short CTI condition (*M* = 30 ms). This effect pattern is in line with the assumption that the utilization of reward information improves with increasing CTI duration. Furthermore, we obtained a marginally significant three-way interaction of the factors reward, CTI, and SOA, *F*(2, 48) = 3.03, *p* = 0.058, η_G_^2^ = 0.003. That showed a trend towards larger reward effects for SOA 100 in the long CTI condition, compared with the short CTI condition. The interaction of the factors SOA and CTI, *F*(2, 48) = 0.18, *p* = 0.840, η_G_^2^ < 0.001, did not reach significance.

#### Effects on the distribution of RT1

To further elucidate the temporal dynamics of reward utilization, we conducted a distribution analysis of RT1. Our reasoning focused on the potential performance improvements at the different tails of the RT distribution. Here we assumed, that in trials with shorter RTs (i.e., the left tail), cognitive processes are optimally executed and thus no further or only slight improvements are obtainable by the prospect of reward, irrespective of the CTI condition. In contrast, during trials with longer RTs (i.e., the right tail), the prospect of reward might further optimize the cognitive processing chain. Such an effect pattern might be more pronounced in the long CTI compared with the short CTI condition, as the effect of reward prospect might evolve leading to increased reward effects on RT1.

**Fig. 3:**
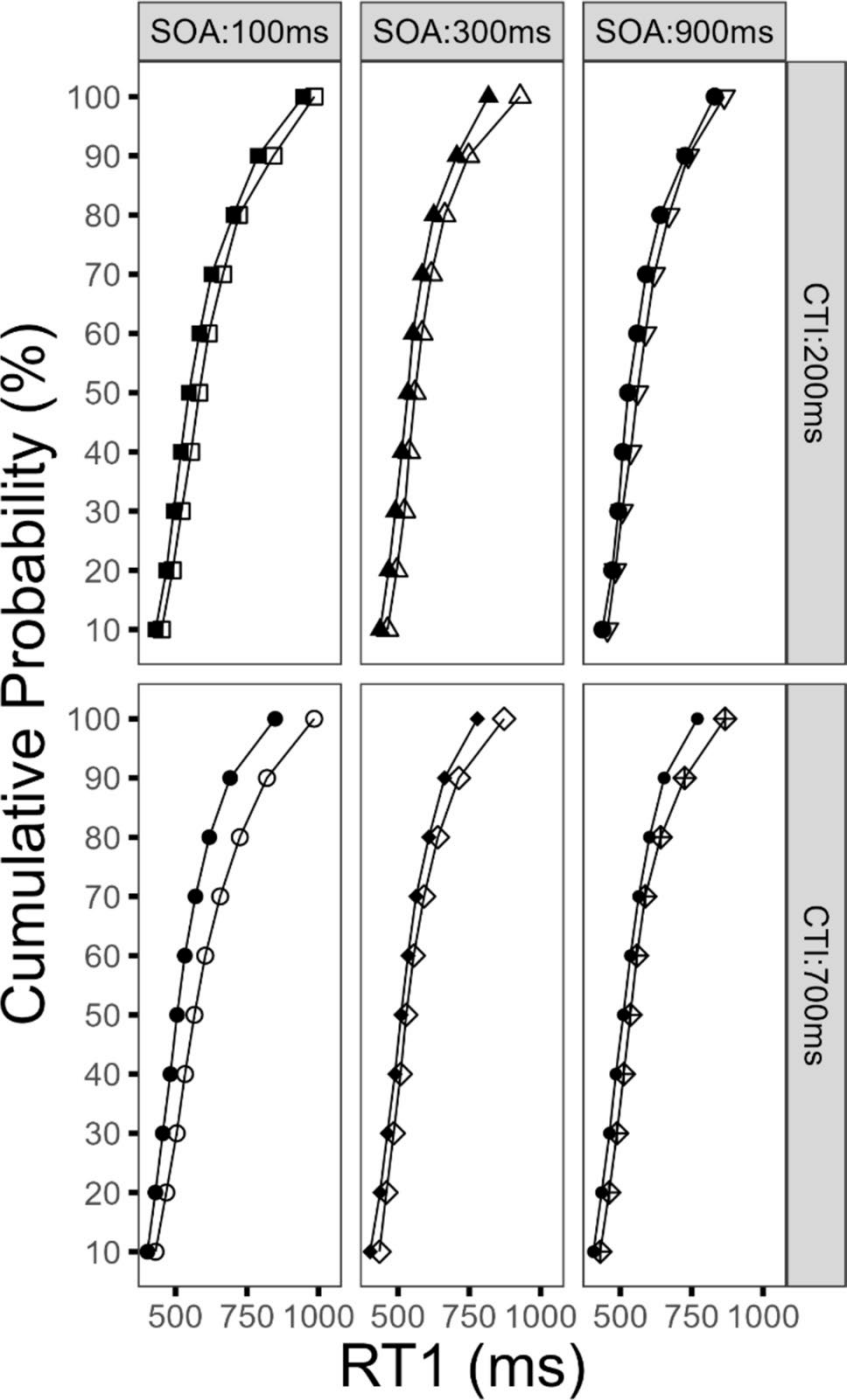
Analysis of reaction time distribution for Task 1 (RT1) as a function of percentile, reward, stimulus-onset asynchrony (SOA), and cue–target interval (CTI) for Experiment 1. Filled symbols denote the reward condition. Empty symbols denote the no-reward condition

For this purpose, the RT1 was rank-ordered from slowest to fastest. Subsequently, the means were computed for each factor combination and RT1 was corrected for outliers (± 2.5 *SD*). After that, the percentiles (10 equal bins) for each factor combination were computed based on the outlier corrected RT1, and subsequently the means were calculated (Schubert, 1999). The mean RT1 for the respective factor combination is plotted on the *x*-axis, while the cumulative distribution is plotted on the *y*-axis (i.e., the respective percentile from 1 to 10 (see Fig. 3). For statistical computations, the factor percentile was used along with the factors reward, SOA, and CTI in an ANOVA. However, to avoid redundancy, we will report only the statistical parameters for effects that include the factor *percentile*. This decision is based on the corresponding results of the previously reported analysis of mean RTs and the results of the current distributional analysis.

First, we obtained a three-way interaction of the factors reward, CTI, and percentile, *F*(9, 216) = 2.01, *p* < 0.039, η_G_^2^ = 0.002. As depicted in Fig. 3 the reward effects were larger in the long CTI compared with the short CTI condition. This pattern was further specified as the reward effects increased with increasing RT1. This effect indicates that the reward effect increased with a longer CTI and that longer RTs were affected in particular. In sum, this suggests that during trials with longer RTs, the impact of the reward prospect in the long CTI condition on cognitive processes was increased.

In addition, the reward effects increased as RT1 got slower. This observation was confirmed by a significant interaction of the factors reward and percentile on RT1, *F*(9, 216) = 6.73, *p* < 0.001, η_G_^2^ = 0.007. Furthermore, the factor percentile reached significance, *F*(9, 216) = 229.01, *p* < 0.001, η_G_^2^ = 0.591. The interaction of the factors SOA and percentile reached also significance, *F*(18, 432) = 12.83, *p* < 0.001, η_G_^2^ = 0.022.

Neither of the following interactions reached significance. The interaction of the factors percentile and CTI did not reach significance, *F*(9, 216) = 1.39, *p* = 0.192, η_G_^2^ = 0.001. Similarly, the interaction of the factors percentile, reward, and SOA was nonsignificant, *F*(18, 432) = 0.99, *p* = 0.466, η_G_^2^ = 0.001. The interaction of the factors percentile, CTI, and SOA did not reach significance, *F*(18, 432) = 0.11, *p* = 0.984, η_G_^2^ < 0.001. The four-way interaction of the factors percentile, reward, CTI, and SOA did not reach significance, *F*(18, 432) = 0.77, *p* = 0.738, η_G_^2^ < 0.001.

For the error rates in Task 1, we obtained neither a significant main effect nor a significant interaction (see Table 1). The main effect of the factor reward did not reach significance, *F*(1, 24) = 2.22, *p* = 0.149, η_G_^2^ = 0.004. Furthermore, the interaction of the factors reward and CTI also did not reach significance, *F*(1, 24) = 1.69, *p* = 0.206, η_G_^2^ = 0.003. Furthermore, the three-way interaction of the factors reward, CTI, and SOA did not reach significance, *F*(2, 48) = 1.69, *p* = 0.195, η_G_^2^ = 0.008. Furthermore, the factor SOA did not reach significance, *F*(2, 48) = 1.06, *p* = 0.356, η_G_^2^ = 0.006. The factor CTI did not reach significance, *F*(1, 24) = 0.01, *p* = 0.922, η_G_^2^ < 0.001. The interaction of the factors reward and SOA also did not reach significance, *F*(2, 48) = 0.22, *p* = 0.806, η_G_^2^ = 0.001.

**Table 1:** Mean rates of errors for Task 1 and Task 2 in % (and standard error of the mean) from Experiment 1 as a function of reward, stimulus-onset asynchrony (SOA), and cue–target interval (CTI)

			Experiment 1						
	Reward-CTI								
	Reward – Short CTI		No Reward – Short CTI		Reward – Long CTI			No Reward – Long CTI	
SOA	Task 1	Task 2	Task 1	Task 2	Task 1	Task 2		Task 1	Task 2
100	6.2% (.7%)	2.8% (.5%)	6.1% (.9%)	2.8% (.8%)	6.6% (.9%)	3% (.7%)		5.9% (.9%)	3.0% (.6%)
300	5% (.8%)	3% (.5%)	5.8% (.8%)	3% (.6%)	7.5% (1.1%)	2.8% (.6%)		4.4% (.6%)	2.4% (.5%)
900	5.9% (.9%)	3.6% (.7%)	5% (.9%)	2.7% (.9%)	4.9% (1.1%)		3.1% (.7%)	4.9% (.8%)	2.8% (1%)

#### Task 2

Next, we tested how Task 2 performance was affected by reward. We obtained a significant main effect of the factor reward, *F*(1, 24) = 21.47, *p* < 0.001, η_G_^2^ = 0.025, on RT2. Participants responded faster in the reward (*M* = 635 ms) compared with the no-reward (*M* = 673 ms; see Fig. 2) condition. Furthermore, we found a significant main effect of the factor SOA, *F*(2, 48) = 271.38, *p* < 0.001, η_G_^2^ = 0.683. RT2 increased from SOA 900 (*M* = 438 ms) to SOA 100 (*M* = 867 ms), indicating the typical PRP effect (Pashler, 1994). In addition, we found a significant main effect of the factor CTI, *F*(1, 24) = 7.61, *p* < 0.011, η_G_^2^ = 0.006, on RT2. Participants responded faster in the long CTI condition (*M* = 644 ms) compared with the short CTI condition (*M* = 664 ms), reflecting the temporal preparation effect on RT2 performance (Niemi & Näätänen, 1981).

Furthermore, we obtained a significant overadditive interaction of the factors reward and SOA, *F*(2, 48) = 11.86, *p* < 0.001, η_G_^2^ = 0.007, on RT2. Pair-wise comparisons revealed a significantly larger reward effect for SOA 100 (*M* = 62 ms) compared with SOA 900 (*M* = 13 ms), *t*(24) = 5.175, *p* < 0.001. This overadditive interaction of reward and SOA on RT2 is consistent with the assumption of effect propagation from Task 1 onto Task 2, improving RT2 as well. This is in line with previous evidence, indicating that the prospect of reward affected the pre- and/or bottleneck stages of Task 1 (Langsdorf et al., 2022).

Central for the issue of how reward utilization is affected by the temporal duration of the CTI condition, we obtained a significant overadditive interaction of the factors reward and CTI on RT2, *F(*1, 24) = 9.43, *p* < 0.005, η_G_^2^ = 0.003. We obtained larger reward effects in the long CTI condition (*M* = 51 ms) compared with the short CTI condition (*M* = 26 ms). We further obtained a trend for a significant three-way interaction of the factors reward, CTI, and SOA on RT2, *F*(2, 48) = 2.63, *p* = 0.083, η_G_^2^ = 0.002. This effect hints at larger reward effects in the long CTI condition at SOA 100 compared with the reward effects at SOA 100 in the short CTI condition. Taken together, these effects are consistent with the assumption that the utilization of reward information improves with increasing CTI duration.

Finally, we obtained an overadditive interaction of the factors CTI and SOA, *F*(2, 48) = 7.09, *p* < 0.002, η_G_^2^ = 0.004. Further tests indicated a larger CTI effect (*M* = 31 ms) for SOA 100 compared with SOA 900 (*M* =  − 3 ms), *t*(24) = 3.208, *p* < 0.001. This result indicates that temporal preparation affected the processing stages before or/at the bottleneck of Task 1, leading to effect propagation onto Task 2, affecting also RT2 (Bausenhart et al., 2006).

#### Effects on the distribution of RT2

The subsequent analysis focused on the temporal dynamics of reward utilization and the observable effects on the distribution of RT2. For this purpose, we conducted a Vincentized distribution analysis (identical procedure as for Task 1) and added the factor percentile to the factors reward, SOA, and CTI in an ANOVA. In order to avoid redundancy, we will report only the statistical parameters for effects that include the factor *percentile*, for the previously indicated reason.

**Fig. 4:**
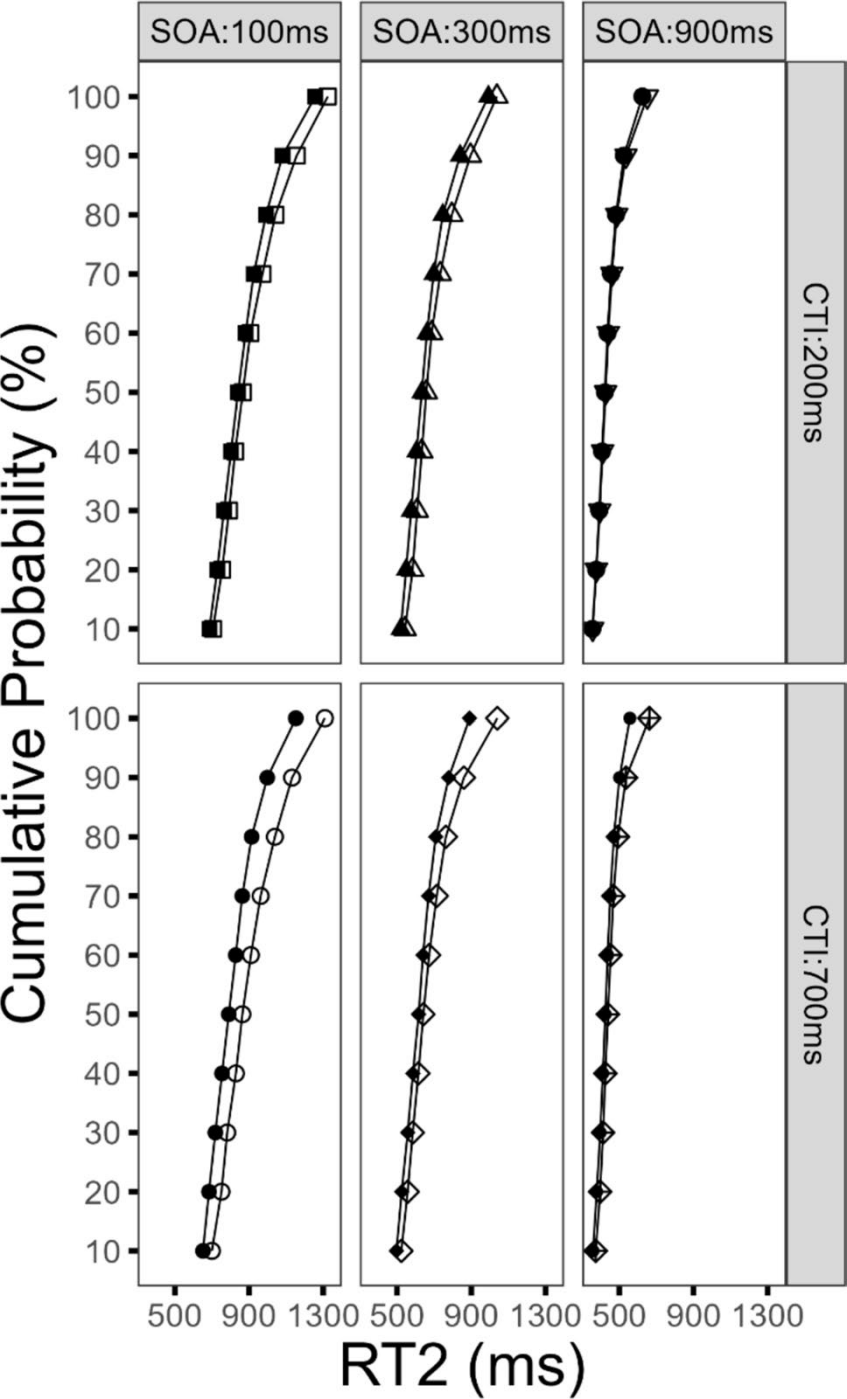
Analysis of reaction time distribution for Task 2 (RT2) as a function of percentile, reward, stimulus-onset asynchrony (SOA), and cue–target interval (CTI) for Experiment 1. Filled symbols denote the reward condition. Empty symbols denote the no-reward condition

First, we obtained a significant three-way interaction of the factors reward, CTI, and percentile, *F*(9, 216) = 3.31, *p* < 0.031, η_G_^2^ = 0.001. As depicted in Fig. 4, the reward effects increased in the long CTI compared with the short CTI condition. Furthermore, these reward effects increased with increasing RT2. This effect demonstrates that the reward effect increased with a longer CTI and that longer RTs were affected in particular. As a result, such an effect pattern could indicate that for trials with longer RTs the impact of the reward prospect on the cognitive processing chain was more effective in the long CTI condition, compared with the short CTI condition.

In addition, the reward effects increased as RT2 got slower. This observation was verified by a significant interaction of the factors reward and percentile on RT2, *F*(9, 216) = 7.94, *p* < 0.001, η_G_^2^ = 0.006. Furthermore, the factor percentile reached significance, *F*(9, 216) = 278.29, *p* < 0.001, η_G_^2^ = 0.450. The interaction of the factors SOA and percentile also reached significance, *F*(18, 432) = 64.92, *p* < 0.001, η_G_^2^ = 0.076. In addition, we obtained a significant interaction of the factors percentile and CTI, *F*(9, 216) = 5.30, *p* < 0.001, η_G_^2^ = 0.002.

Neither of the following interactions reached significance. The interaction of the factors percentile, reward, and SOA was nonsignificant, *F*(18, 432) = 0.99, *p* = 0.470, η_G_^2^ < 0.001. The interaction of the factors percentile, CTI, and SOA did not reach significance, *F*(18, 432) = 0.11, *p* = 0.999, η_G_^2^ < 0.001. Finally, the interaction of the factors percentile, reward, CTI, and SOA was not significant, *F*(18, 432) = 0.32, *p* = 0.997, η_G_^2^ = 0.001.

For the error rates on Task 2, we obtained neither a significant main effect nor a significant interaction. The factor reward did not reach significance, *F*(1, 24) = 1.37, *p* = 0.253, η_p_^2^ = 0.001. In addition, the interaction of the factors reward and CTI did not reach significance, *F*(1, 24) = 0.03, *p* = 0.871, η_p_^2^ < 0.001. Furthermore, the interaction of the factors SOA, reward, and CTI did not reach significance, *F*(2, 48) = 0.39, *p* = 0.682, η_p_^2^ < 0.001. The main effect of the factor CTI did not reach significance, *F*(1, 24) = 0.29, *p* = 0.596, η_p_^2^ < 0.001. Furthermore, the effect of the factor SOA did not reach significance, *F*(2, 48) = 0.10, *p* = 0.903, η_p_^2^ < 0.001. The interaction of the factors reward and SOA did not reach significance, *F*(2, 48) = 0.36, *p* = 0.701, η_G_^2^ < 0.001. The interaction of the factors SOA and CTI did not reach significance, *F*(2, 48) = 0.19, *p* = 0.828, η_p_^2^ < 0.001.

## Discussion

In Experiment 1, participants were able to flexibly utilize the reward information on a trial-to-trial basis to improve their DT performance, as reflected by the main effects of reward on RT1 and RT2. Furthermore, we obtained an overadditive interaction of reward and SOA on RT2, with larger reward effects at short compared with long SOA. These findings are consistent with the assumption that reward affected pre- and/or bottleneck stages of Task 1 improving RT1. As a result, the reward-related processing improvements propagated onto Task 2 at short SOA via the bottleneck mechanism to improve RT2, while for the long SOA condition, no bottleneck emerges preventing the transmission between tasks. This effect pattern suggests that participants were able to flexibly utilize reward information for behavioral DT improvements (Langsdorf et al., 2022).

Importantly, we furthermore obtained results in line with the assumption that the utilization of reward information depends on the temporal duration of the preparatory interval. This is reflected by the overadditive interaction of reward and CTI on mean RT1 and RT2, with larger reward effects in the long CTI compared with the short CTI condition. These results were further substantiated by the results of the RT distribution analysis, with increased reward effects in the long CTI compared with the short CTI condition on RT1 and RT2, especially on longer RTs. These effects indicate that the joint effect of reward prospect and CTI more efficiently optimizes longer RTs in the long CTI condition. Furthermore, these effects where accompanied by a main effect of CTI on RT1 and RT2, reflecting optimized preparation. Consequently, we obtained a combined effect of enhanced reward utilization and improved task preparation. We will come back to these points in the General Discussion section.

For Experiment 2, we aimed to test whether temporal expectation affects the temporal dynamics of reward utilization in Experiment 1. Therefore, we applied the CTIs randomized within blocks, as this reduces the temporal expectation of participants for target onset (Fischer et al., 2007). In contrast, for Experiment 1, a blocked presentation of CTI was applied which should have led to a precise temporal expectation of Task 1 onset by the participants. As a result, the temporal expectation might have affected the temporal dynamics of reward utilization in Experiment 1.

## Experiment 2

In Experiment 2, we investigated the temporal dynamics of reward utilization for DT conditions in which participants cannot build up a precise temporal expectation of Task 1 onset. This enabled us to investigate whether temporal expectation affects the temporal dynamics of reward utilization. To this end, we combined a trial-wise reward prospect for participants' Task 1 performance, indicated by a cue signaling either a reward or no-reward trial with a randomized CTI of either 200 ms or 700 ms. Thus, for both CTI conditions, a cue was presented signaling either a reward or no-reward trial. The randomized CTI application increased the temporal uncertainty of the participants, as either a CTI of 200 ms or 700 ms could be presented. Finally, the task situation comprised of an auditory-visual DT with three SOAs (100 ms, 300 ms, or 900 ms). The reward application was identical to Experiment 1 as we instructed participants that they could earn a monetary reward if their response to Task 1 was fast and accurate while minding a low error rate in Task 2.

### Materials and methods

#### Participants

Twenty-five healthy participants (20 women; *M*_age_ = 23 years) were invited to take part in the experiment. One participant had to be excluded due to technical difficulties. The further procedure was identical to Experiment 1.

#### Apparatus and stimuli

The apparatus and stimuli were identical to Experiment 1. The only exception was that within each DT block the CTIs of 200 ms or 700 ms were randomly presented.

#### Design and procedure

The design and procedure were identical to Experiment 1.

#### Statistical analysis

All analyses on the RTs and error rates for Task 1 and Task 2 were identical to Experiment 1. For the RT analyses, trials with at least one erroneous response (*M* = 8.3%) and outliers that deviated more than ± 2.5 standard deviations for each participant and factor combination (*M* = 4.6*%*) were excluded from the data set.

### Results

#### Task 1

**Fig. 5:**
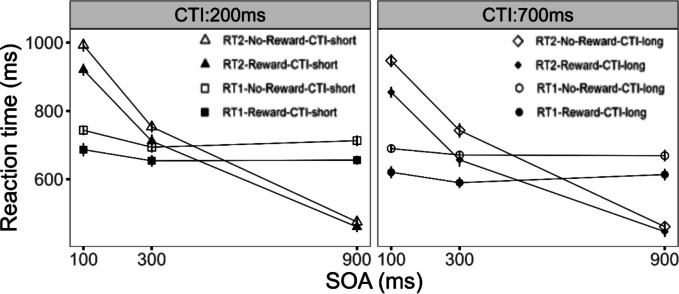
Mean RTs for Task 1 and Task 2 as a function stimulus-onset asynchrony (SOA), and cue–target interval (CTI) for Experiment 2. Error bars represent the standard error of the mean

We first tested for the effects of reward on Task 1 performance. We obtained a significant main effect of the factor reward, *F*(1, 23) = 23.20, *p* < 0.001, η_G_^2^ = 0.037. Participants’ RT1 was reduced in the reward (*M* = 634 ms) compared with the no-reward (*M* = 696 ms; see Fig. 5) condition. Furthermore, we obtained a significant main effect of the factor SOA on RT1, *F*(2, 46) = 5.76, *p* < 0.006, η_G_^2^ = 0.008, with increasing RT1 for shorter SOAs. These effects of SOA on Task 1 performance are usually explained with strategical response grouping by the participants (Strobach et al., 2015; Ulrich & Miller, 2008). In addition, we obtained a significant main effect of the factor CTI, *F*(1, 23) = 68.77, *p* < 0.001, η_G_^2^ = 0.025. Participants´ RT1 was reduced in the long CTI condition (*M* = 640 ms) compared with the short CTI condition (*M* = 686 ms), indicating the temporal preparation effect on RT1 (Niemi & Näätänen, 1981).

Most importantly, for the issue of the temporal dynamics of reward utilization, we obtained a significant overadditive interaction of the factors reward and CTI, *F*(1, 23) = 5.12, *p* < 0.033, η_G_^2^ < 0.001, on RT1. The reward effect was increased in the long CTI (*M* = 69 ms) compared with the short CTI (*M* = 51 ms) condition. As a result, this effect pattern is in line with the assumption that the utilization of reward information improves with increasing processing duration for DT conditions of increased temporal uncertainty.

Neither the interaction of the factors reward and SOA reached significance, *F*(2, 46) = 0.34, *p* = 0.711, η_G_^2^ < 0.001, nor the interaction of the factors, SOA and CTI, *F*(2, 46) = 1.11, *p* = 0.338, η_G_^2^ < 0.001. Finally, we did not obtain a significant three-way interaction of the factors reward, CTI, and SOA, *F*(2, 46) = 1.48, *p* = 0.239, η_G_^2^ < 0.001.

#### Effects on the distribution of RT1

As for Experiment 1, we conducted a Vincentized distribution analysis of RT1 to further investigate the temporal dynamics of reward utilization. The procedure to compute the distribution analysis was identical to the procedure described in Experiment 1. The resulting factor percentile was added along with the factors reward, SOA, and CTI in an ANOVA. With the aim of avoiding redundancy, we will report only the statistical parameters for effects that include the factor *percentile* for the previously indicated reason.

**Fig. 6:**
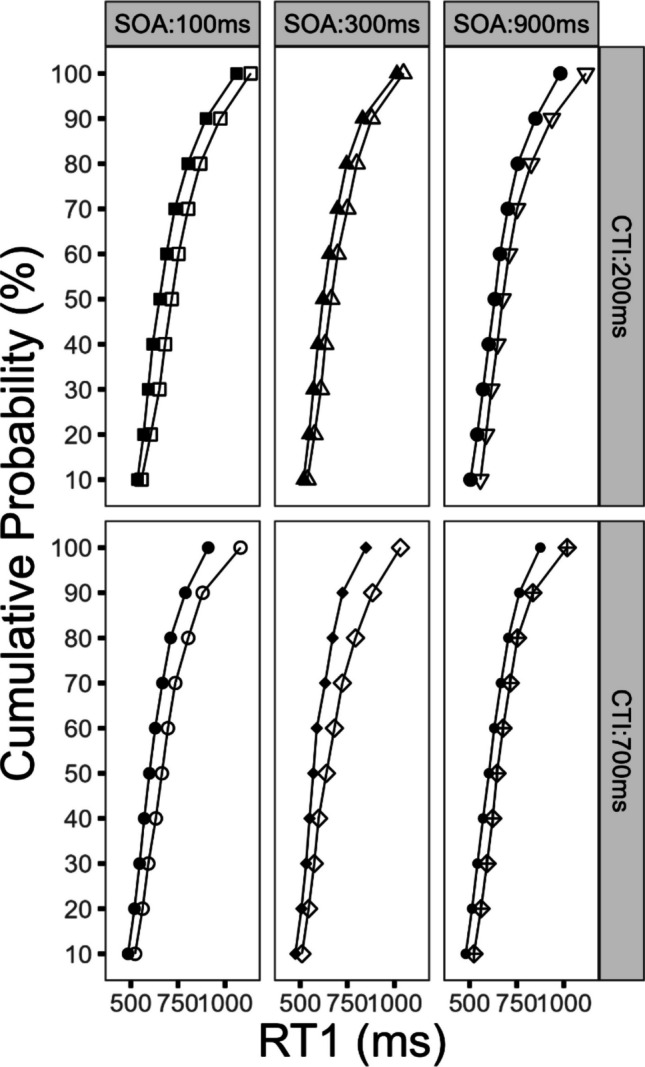
Analysis of reaction time distribution for Task 1 (RT1) as a function of percentile, reward, stimulus-onset asynchrony (SOA), and cue–target interval (CTI) for Experiment 2. Filled symbols denote the reward condition. Empty symbols denote the no-reward condition

First, we obtained a significant three-way interaction of the factors reward, CTI, and percentile, *F*(9, 207) = 2.33, *p* < 0.016, η_G_^2^ = 0.002. As depicted in Fig. 6, the reward effects increased for the long CTI compared with the short CTI condition. This effect was further specified, as the reward effect increased with increasing RT1. This result indicates increased reward effects in the long compared with the short CTI condition, with a stronger benefit for slower RTs, for DT conditions of increased temporal uncertainty.

In addition, the reward effect increased with longer RT1, which is confirmed by the interaction of the factors reward and percentile, *F*(9, 207) = 8.11, *p* < 0.001, η_G_^2^ = 0.005. The reward effect pattern demonstrates that the prospect of reward affects specifically longer RTs. We also obtained a significant main effect of the factor percentile, *F*(9, 207) = 103.47, *p* < 0.001, η_G_^2^ = 0.374, and a significant interaction of the factors percentile and CTI, *F*(9, 207) = 9.62, *p* < 0.001, η_G_^2^ = 0.003.

None of the following interactions reached significance. The interaction of the factors percentile, reward, and SOA did not reach significance *F*(18, 414) = 1.12, *p* = 0.325, η_G_^2^ < 0.001. The interaction of the factors percentile, CTI, and SOA did not reach significance, *F*(18, 414) = 0.67, *p* = 0.846, η_G_^2^ = 0.202.The interaction of the factors percentile and SOA did not reach significance, *F*(18, 414) = 1.27, *p* < 0.202, η_G_^2^ = 0.001.The interaction of the factors percentile, reward, CTI, and SOA did not reach significance, *F*(18, 414) = 1.12, *p* = 0.329, η_G_^2^ < 0.001.

For the error rates on Task 1 (see Table 2), we only obtained a significant main effect of the factor SOA, *F*(1, 23) = 8.44, *p* < 0.001, η_G_^2^ = 0.015, with increased error rates for SOA 100 (*M* = 7%) compared with SOA 300 (*M* = 5%) and SOA 900 (*M* = 5%). The factor reward did not reach significance, *F*(1, 23) = 1.19, *p* = 0.287, η_G_^2^ = 0.003. The interaction of the factors reward and CTI did not reach significance, *F*(1, 23) = 0.04, *p* = 0.842, η_p_^2^ < 0.001. Also, the three-way interaction of the factors reward, SOA, and CTI did not reach significance, *F*(2, 46) = 0.07, *p* = 0.936, η_p_^2^ < 0.001. The interaction of the factors reward and SOA did not reach significance, *F*(2, 46) = 0.01, *p* = 0.980, η_p_^2^ < 0.001. In addition, the interaction of the factors SOA and CTI did not reach significance, *F*(2, 46) = 0.39, *p* = 0.679, η_p_^2^ < 0.001.

**Table 2:** Mean rates of errors for Task 1 and Task 2 in % (and standard deviation) from Experiment 2 as a function of reward, stimulus-onset asynchrony (SOA), and cue–target interval (CTI)

			Experiment 2						
	Reward-CTI								
	Reward – Short CTI		No Reward – Short CTI		Reward – Long CTI			No Reward – Long CTI	
SOA	Task 1	Task 2	Task 1	Task 2	Task 1	Task 2		Task 1	Task 2
100	6.3% (1.3%)	3.2% (.9%)	7.3% (1%)	2.2% (.5%)	7.1% (1%)	3.4% (.8%)		7.7% (1.0%)	2% (.5%)
300	5.2% (.7%)	2.3% (.6%)	5.9% (.9%)	2.3% (.7%)	4.2% (1.2%)	.9% (.5%)		5.4% (.7%)	2.4% (.7%)
900	4.5% (.7%)	4% (.7%)	5.4% (.8%)	3.4% (.6%)	4.6% (.8%)		1.9% (.4%)	5.5% (.8%)	2.7% (.8%)

#### Task 2

Next we tested for the effects of reward on Task 2 performance**.** We observed a significant main effect of the factor reward on RT2, *F*(1, 23) = 52.22, *p* < 0.001, η_G_^2^ < 0.019. Participants responded faster in the reward (*M* = 677 ms) compared with the no-reward condition (*M* = 728 ms; see Fig. 5). Furthermore, the factor SOA reached significance, *F*(2, 46) = 343.12, *p* < 0.001, η_G_^2^ < 0.498. RT2 increased from SOA 900 (*M* = 462 ms) to SOA 100 (*M* = 927 ms) demonstrating the typical PRP effect (Pashler, 1994). In addition, we obtained a significant main effect of the factor CTI, *F*(1, 23) = 45.93, *p* < 0.001, η_G_^2^ < 0.008. Participants responded faster in the long CTI condition (*M* = 683 ms) compared with the short CTI condition (*M* = 717 ms), demonstrating the temporal preparation effect on RT2 (Niemi & Näätänen, 1981).

Most importantly, for the issue of the temporal dynamics of reward utilization, we obtained a significant overadditive interaction of the factors reward and CTI, *F*(1, 23) = 4.89, *p* < 0.037, η_G_^2^ = 0.001, on RT2. The reward effects increased in the long CTI (*M* = 64 ms) compared with the short CTI (*M* = 43 ms) condition. This effect is consistent with the assumption that reward utilization improves with increasing CTI for DT conditions of reduced temporal expectation. Furthermore, the three-way interaction of the factors reward, CTI, and SOA did not reach significance, *F*(2, 46) = 2.20, *p* = 0.122, η_G_^2^ < 0.001.

Furthermore, we obtained a significant overadditive interaction of the factors reward and SOA, *F*(2, 46) = 19.07, *p* < 0.001, η_G_^2^ < 0.006, with larger reward effects at SOA 100 (*M* = 82 ms) compared with SOA 900 (*M* = 15 ms), *t*(23) = 6.371, *p* < 0.001 (Langsdorf et al., 2022). In addition, we obtained a significant overadditive interaction of the factors CTI and SOA, *F*(2, 46) = 3.34, *p* = 0.044, η_G_^2^ < 0.002. The CTI effect was increased for SOA 100 (*M* = 56 ms) compared with SOA 900 (*M* = 14 ms), *t*(23) = 2.724, *p* < . 012 (Bausenhart et al., 2006). These effects are in line with the results of Experiment 1 and the remarks from the introduction section, suggesting a pre-motoric locus of the reward and temporal preparation effect on Task 1 processes.

#### Effects on the distribution of RT2

In addition, we conducted a Vincentized distribution analysis of RT2 to investigate the temporal dynamics of reward utilization in more detail. The procedure to compute the distribution analysis was identical to the procedure described for Experiment 1. Subsequently, the factors percentile, reward, SOA, and CTI were used in an ANOVA. With the aim of avoiding redundancy, we will report only the statistical parameters for effects that include the factor *percentile* for the previously indicated reason.

**Fig. 7:**
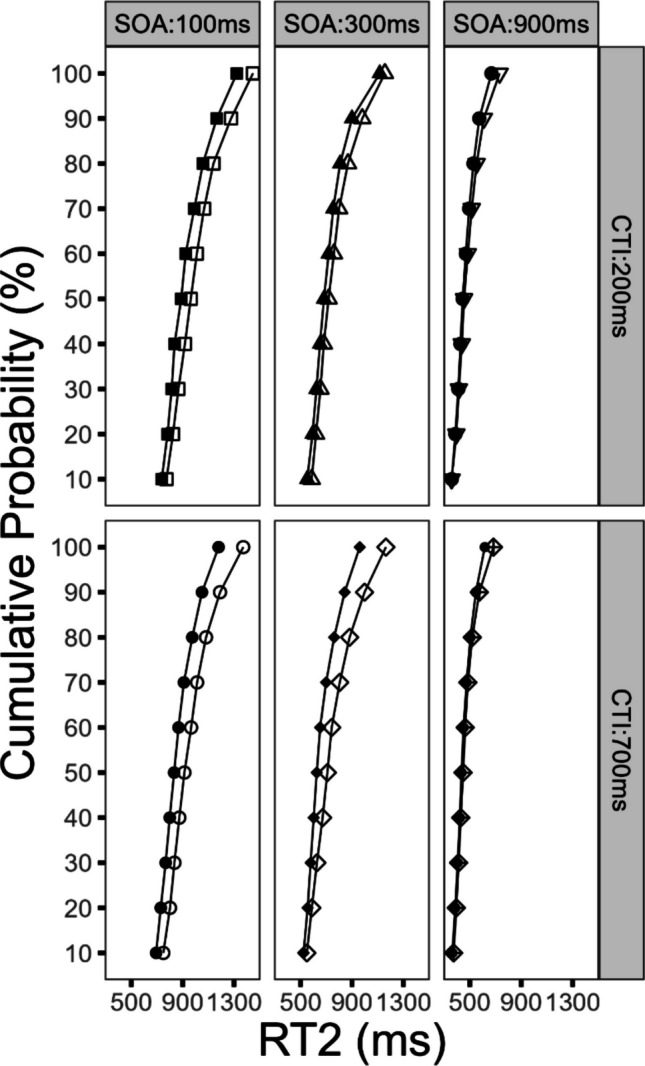
Analysis of reaction time distribution for Task 2 (RT2) as a function of percentile, reward, stimulus-onset asynchrony (SOA), and cue–target interval (CTI) for Experiment 2. Filled symbols denote the reward condition. Empty symbols denote the no-reward condition

First, we obtained a significant three-way interaction of the factors reward, CTI, and percentile, *F*(9, 207) = 2.50, *p* < 0.010, η_G_^2^ = 0.001. As depicted in Fig. 7, the reward effects in the long CTI compared with the short CTI condition were increased. This effect was further specified as the reward effects further increased, as RT2 got slower. In sum, this finding indicates that the reward effects increased with an increasing CTI, while longer RTs seemed to be especially susceptible to processing improvements.

Furthermore, the reward effects increased as RT2 increased. This observation was confirmed by a significant interaction of the factors reward and percentile on RT2, *F*(9, 207) = 16.04, *p* < 0.001, η_G_^2^ = 0.003. In addition, we obtained several further findings; the factor percentile reached significance, *F*(9, 207) = 60.63, *p* < 0.001, η_G_^2^ = 0.278. The interaction of the factors SOA and percentile also reached significance, *F*(18, 414) = 60.00, *p* < 0.001, η_G_^2^ = 0.027. In addition, we obtained a significant interaction of the factors percentile and CTI, *F*(9, 207) = 5.52, *p* < 0.001, η_G_^2^ = 0.001.

None of the following interactions reached significance: the interaction of the factors percentile, reward, and SOA did not reach significance, *F*(18, 414) = 1.15, *p* = 0.305, η_G_^2^ < 0.001. Similarly, the interaction of the factors percentile, CTI, and SOA was not significant, *F*(18, 414) = 1.31, *p* = 0.174, η_G_^2^ < 0.001. In addition, the four-way interaction of the factors percentile, reward, CTI, and SOA did not reach significance, *F*(18, 414) = 1.34, *p* = 0.161, η_G_^2^ < 0.001.

For the error rates on Task 2, only the interaction of the factors reward and SOA reached significance, *F*(2, 46) = 3.36, *p* < 0.044, η_G_^2^ = 0.015. Further tests indicated that there was a trend towards a slightly increased error rate for SOA 100, in the reward (*M* = 3.3%) compared with the no-reward condition (*M* = 2.1%), *t*(23) = 1.88, *p* = 0.072. This was not the case for the SOA 300 and SOA 900 conditions, as the descriptive data indicates a reduced error rate in the reward compared with the no-reward condition. As a result, the slightly increased error rate in the reward SOA 100 condition was accompanied by a reduced RT2; together these effects could suggest a dynamic adjustment of the response criteria of the participants.

Furthermore, the factor SOA did not reach significance, *F*(2, 46) = 2.18, *p* = 0.125, η_G_^2^ = 0.015. In addition, the interaction of the factors reward and CTI showed a trend, *F*(1, 23) = 3.59, *p* = 0.071, η_G_^2^ = 0.005. The main effect of the factor CTI did not reach significance, *F*(1, 23) = 3.00, *p* = 0.097, η_G_^2^ = 0.004. Also the interaction of the factors SOA and CTI did not reach significance, *F*(2, 46) = 0.97, *p* = 0.385, η_p_^2^ = 0.007. The main effect of the factor reward did not reach significance, *F*(1, 23) = 0.04, *p* = 0.835, η_G_^2^ < 0.001. In addition, the three-way interaction of the factors SOA, reward, and CTI did not reach significance, *F*(2, 46) = 0.83, *p* = 0.442, η_G_^2^ = 0.002.

#### Discussion

In Experiment 2, the trial-wise reward application for Task 1 performance resulted in a replication of the reward-related processing improvements from Experiment 1, as reflected by the main effect of reward on RT1 and RT2, and by the overadditive interaction of reward and SOA on RT2.

Importantly, the obtained results favor the assumption that the utilization of reward information improves with increasing CTI duration, as we obtained an overadditive interaction of reward and CTI on RT1 and RT2, with larger reward effects in the long CTI compared with the short CTI condition. This effect pattern was further specified by the results of a distribution analysis of RTs, indicating larger reward effects on longer RTs. Again, these reward-related processing improvements were accompanied by a main effect of CTI on RT1/RT2, reflecting improved task preparation. As a result, optimized task processing was further optimized by enhanced reward utilization, also for DT condition of increased temporal uncertainty. We will come back to this point in the general discussion part.

## General discussion

The present study investigated whether participants can flexibly utilize trial-wise reward information to improve their DT performance. For this purpose, in Experiment 1, we applied a trial-wise reward application for participants' Task 1 performance in a PRP DT situation. We obtained an improved RT1 performance in the reward compared with the no-reward condition. Furthermore, we obtained an overadditive interaction of reward and SOA on RT2, with larger reward effects at short compared with long SOA. According to the effect propagation logic, such a reward pattern indicates that the prospect of reward affected the processing stages of Task 1 before or/at the bottleneck leading to a reduction of RT1. As a result, for the short SOA condition, the reward effect was propagated via the bottleneck mechanism onto Task 2, reducing RT2. In contrast for the long SOA condition, no bottleneck emerges between the tasks, thus no reward effect transmission could occur.

The second aim of Experiment 1 was to investigate the temporal dynamics of reward utilization. To this end, we combined a trial-wise reward prospect for Task 1 performance with a blocked CTI of either 200 ms or 700 ms. We obtained an overadditive interaction of reward and CTI on mean RT1 and RT2, with larger reward effects in the long compared with the short CTI condition. This reward pattern was further specified by the results of an RT distribution analysis, with larger reward effects on longer RTs in the long compared with the short CTI condition for RT1 and RT2. This finding suggests that the cognitive processing in trials with longer RTs is particularly susceptible to reward-CTI optimizations. In sum, these results are in line with the assumption that reward utilization is susceptible to the temporal duration of the CTI interval.

For Experiment 2, we investigated how temporal expectation affects the temporal dynamics of reward utilization by presenting varying CTIs randomly within blocks. We obtained an overadditive interaction of reward and CTI on mean RT1 and RT2, with larger reward effects in the long CTI compared with the short CTI condition. These results were further specified by the results of an RT distribution analysis on RT1 and RT2, indicating larger reward effects in the long CTI compared with the short CTI condition on longer RTs. Taken together the results favor the assumption that reward utilization improves with increasing duration of the CTI interval, for DT conditions of reduced temporal expectation.

### Flexible utilization of reward information for behavioral adjustments in dual-task situations

The current study investigated whether participants can flexibly utilize reward information for performance improvements in DT situations. The results indicated that the prospect of reward rapidly improves mean Task 1 and Task 2 performance on a trial-to-trial basis, which suggests a flexible utilization of the reward information. In addition, the reward effects on the mean RT1/RT2 were further specified by larger reward effects on longer RTs, as indicated by the results of the RT1 and RT2 distribution analysis. Former studies reported that increased mental effort can lead to improved attentional mobilization which results in the stabilization of task performance, as was shown by the effect of mental effort on the right tail of the RT distribution (see also Steinborn et al., 2017). The current result pattern is therefore consistent with the assumption that the prospect of reward stabilized DT performance, by reducing the attentional fluctuation of DT performance in the reward compared with the no-reward condition. Furthermore, the current results indicate that substantial performance improvements are obtainable for DT conditions in which 50% of trials required the participants to utilize reward information for performance improvements. Consequently, these results extend previous studies in which a 20/80 proportion of effort and standard trials had been applied, indicating a large adaptivity of reward utilization of participants (see Kleinsorge, 2001; Steinborn et al., 2017; Strayer et al., 2024). Taken together, these results suggest that participants can rapidly utilize the reward information to improve and stabilize their DT performance.

Furthermore, the findings of a flexible utilization of reward information extend previous results with a block-wise reward application in DT situations. In particular, in previous investigations of our group, the application of reward prospect for Task 1 was implemented at the block level. For that purpose, participants were instructed that an entire block was rewarded and each trial was reward-relevant, which led to a constant prospect of reward across the whole rewarded blocks. As a result, participants could develop and apply a strategy of reward-induced preparation throughout the rewarded blocks to adjust their DT performance for obtaining a reward (Langsdorf et al., 2022). This resulted in reward effects on RT1 and larger reward effects at short compared with long SOA on RT2. This effect was interpreted with the effect propagation logic (Pashler, 1994; Schubert, 1999; Schweickert, 1980) indicating that the prospect of reward for Task 1 affected the processing stages of Task 1 before or/at the bottleneck, which was then transmitted via the bottleneck mechanism at short SOA onto the processing chain of Task 2, reducing RT2 as well. In contrast for the long SOA condition, the temporal overlap of the processing chain of Task 1 and Task 2 is reduced preventing the emergence of a bottleneck, thus leading to no effect propagation from Task 1 onto Task 2. In sum, the results of the trial-wise and block-wise reward application for Task 1 performance revealed similar reward effect patterns: the prospect of reward affects the pre-motoric processing stages of Task 1 improving RT1, leading to a transmission of the reward effects onto Task 2, resulting in larger reward effects for short compared with long SOA on RT2.

The pre-motoric locus of the reward effect would be in line with findings indicating that the prospect of reward enhances attentional allocation, stimulus processing, as well as attentional preparation. In particular, in a recent study applying electrophysiology with high temporal resolution, the effect of a cued reward prospect on conflict processing was investigated (Van Den Berg et al., 2014). The results indicated that the reward cue led to an enhancement of neural correlates of attentional allocation, stimulus processing, and attentional preparation. This was reflected by an enhanced amplitude of the N2 a component associated with the allocation of attention to salient stimuli, as well as an enhanced amplitude of the N1 a component associated with stimulus processing. Furthermore, the reward prospect boosted the amplitude of the contingent negative variation (CNV), an indicator of attentional preparation (Schevernels et al., 2014). Taken together, such effects of the reward prospect on neurophysiological components would be in line with our finding of a pre-motoric locus of the reward effect in the processing chain of Task 1 and Task 2.

### Temporal dynamics of reward utilization in dual-task situations: On the relation of reward-related and preparation-related performance improvements

The second aim of the present study was to investigate the temporal dynamics of reward utilization. In particular, we were interested in whether the utilization of reward information is dependent upon the duration of the CTI. For Experiments 1 and 2, we obtained an overadditive interaction of reward and CTI on RT1 and RT2, reflecting larger reward effects in the long compared with the short CTI condition. Importantly, these effects were further specified by larger reward effects on longer RTs in the long CTI condition compared with the short CTI condition, as indicated by the results of the distribution analysis of RT1 and RT2. This effect pattern indicates that the prospect of reward builds up over time to improve the especially long RTs and that this optimization process is more effective at the long CTI condition compared with the short CTI condition. These results are consistent with the assumption that the utilization of reward information is susceptible to the length of the CTI interval. Taken together, the results show, to the best of our knowledge, a novel effect that provides useful insights into the temporal dynamics of reward processing.

Furthermore, these findings are relevant as there is an ongoing discussion on whether reward-related processing improvements reflect in essence preparation-related processing improvements (Capa et al., 2013; Kleinsorge & Rinkenauer, 2012; Rieger et al., 2021; Zedelius et al., 2012) or whether the prospect of reward leads to additional effects on task performance beyond preparation-related improvements. In particular, it had been reported and argued that the prospect of reward leads to improved preparation resulting in processing improvements as reflected by reduced RTs in the reward compared with the no-reward condition as well as to improved activation of ERP components associated with task-related preparation processes (Schevernels et al., 2014).

In contrast, the current findings suggest a different picture, as we observed an *overadditive* interaction of reward and CTI on DT performance, with larger reward-related processing benefits in the long CTI compared with the short CTI condition. As such, the obtained results indicate that even for optimal preparatory conditions as reflected by the improved RT1 and RT2 performance for the long compared with the short CTI condition, the prospect of reward *further* improved DT performance going beyond the preparation-related performance improvements. Such an effect pattern is not consistent with the assumption that the reward effects on the RTs reflect in essence preparation-related processing improvements (e.g., Rieger et al., 2021; Zedelius et al., 2012). But instead, these results favor the assumption that the prospect of reward elicited an additional effect on DT performance which goes beyond the temporal preparation effect. This inference is also in line with the results of the distribution analysis of RT1 and RT2 suggesting that especially trials with longer RTs profit most from the reward prospect in the long CTI condition. Taken together the results of Experiments 1 and 2 indicate that the prospect of reward can further improve DT performance even for optimal preparatory DT conditions.

An important further question is through which mechanism the overadditive reward-CTI interaction is emerging. One possibility could be that the prospect of reward and temporal preparation affect identical processes leading to the observed outcome. In particular, previous evidence by Seibold et al. (2011) demonstrated that conditions of high in contrast to low temporal preparation improved perceptual processing in a sensory-motor RT task by leading to an earlier onset of sensory information accumulation. When we consider the pre-motoric locus of the reward effect in the processing chain of Task 1 and Task 2 then it is conceivable that the prospect of reward improved processes related to the perception of Task 1. Similarly, for both experiments, the locus of the temporal preparation effect was pre-motoric, as reflected by the main effect of CTI on RT1 and the overadditive interaction of CTI and SOA on RT2, with larger CTI effects at short compared with long SOA on RT2. This finding is in line with previous evidence reporting a pre-motoric locus of the temporal preparation effect (Bausenhart et al., 2006, 2010; Seibold et al., 2011), that led to the specification that the temporal preparation effect impacts the onset of sensory information accumulation. Taken together, the loci of the reward effect and the temporal preparation effect are on pre-motoric processing stages in the processing chain of Task 1 and Task 2. As speculation, one could assume that this effect pattern indicates that both manipulations affected the onset of sensory information accumulation in an overadditive way. Such an assumption would be consistent with the observation that the prospect of reward can enhance auditory processing sensitivity in a sensory-motor RT task leading to improved task performance (Asutay & Västfjäll, 2016). In connection with the current results, the overadditive reward-CTI effects on RTs might result from a combined effect on the onset of sensory information accumulation. All in all, while it is conceivable that the prospect of reward and temporal preparation could jointly affect the onset of sensory information accumulation, further investigations are required to precisely establish the mechanism driving the novel overadditive reward-CTI effect.

A further aspect that should be discussed is whether the applied criteria for obtaining a reward may have affected the current result patterns.^3^ Specifically, participants were instructed that fast and accurate Task 1 performance would be rewarded while the criterion applied for reward attainment was based on either RTs *or* error rates. In Experiment 1, this led to enhanced RT1/RT2 performance without differences in error rates between the reward and no-reward conditions. In Experiment 2, while RT1/RT2 performance was similarly enhanced, there was a slight tendency for increased Task 2 error rates in the reward compared with the no-reward condition for the SOA 100 task situation. These results indicate that our reward criterion instruction for obtaining a reward did not lead to a systematic prioritization of speed over accuracy by the participants.

This conclusion is further corroborated by previous results of our group, in which the same criteria for obtaining a reward led to improved RT1/RT2 processing *and* reduced error rates in the reward compared with the no-reward condition (Langsdorf et al., 2025). In sum, combined evidence suggests that our applied criteria for reward attainment reliably improves processing speed, while varying effects on the error rates can occur (see also Falkenstein et al., 2003; Kleinsorge, 2001). Future studies might systematically investigate how varying criteria for reward attainment affect RTs and error rates for varying task conditions.

### Reward utilization in dual-task situations with reduced temporal expectation

A further relevant aspect was whether temporal expectation affected the utilization of reward information in Experiment 1 as we applied blocked CTIs for which participants could develop a precise temporal expectation of Task 1 onset. This task setup led to an overadditve interaction of reward and CTI on RT1 and RT2, reflecting larger reward effects in the long compared with the short CTI condition. In contrast, for Experiment 2, we applied randomized CTIs for which participants could not develop a precise temporal expectation of Task 1 onset. For this case, temporal uncertainty emerged as it was not evident at the start of the trial which CTI would be presented to the participants. The obtained results indicated that for DT conditions with temporal uncertainty, the reward effects in the long CTI were increased compared with the short CTI condition for RT1 and RT2. This indicates that the utilization of reward information improved for longer preparation durations, also under conditions of less predictable DT situations.

However, it has to be noted that the application of the randomized CTIs from Experiment 2 still enables the participants to establish a temporal expectation of Task 1 onset. As the CTIs were not drawn from a nonaging but from an aging distribution, the conditional probability of Task 1 onset increased with increasing CTI length (Fischer et al., 2007). Based upon that participants may utilize this information to estimate Task 1 onset to strategically prepare for Task 1 processing. Future studies could further control for such strategical preparation effects by drawing the CTIs from a nonaging distribution to explore the boundary conditions for the reward–CTI interaction.

### Current directions in the investigation of reward-related processing improvements in sensory-motor RT tasks

The present investigation aligns with several other studies that explored reward-related processing improvements (Chiew & Braver, 2016; Falkenstein et al., 2003; Fischer et al., 2018; Kleinsorge, 2001; Kleinsorge & Rinkenauer, 2012; Kool & Botvinick, 2018; Langsdorf et al., 2022, 2025; Rieger et al., 2021; Steinborn et al., 2017). In most of these studies, it was assumed that the prospect of reward ramps up task-related preparatory processes, leading to improved task performance. However, accumulating evidence suggests that the prospect of reward could stimulate additional optimization processes.

A further relevant line of research provided evidence consistent with the assumption that the prospect of reward can modulate the flexibility-stability balance of cognitive control (Shen & Chun, 2011; see also Fröber & Dreisbach, 2014, 2016; Fröber et al., 2019). To investigate this, the authors applied the task-switching methodology, in which participants switch (change task compared with previous trial) or repeat (repeat task compared with previous trial) between two sensory-motor RT tasks. This typically leads to longer RTs for the switch compared with the repetition condition, and the resultant switch costs (i.e., switch–repeat RTs) are considered as an indicator of cognitive flexibility. The authors reported that a constant reward prospect from trial *n*−1 to trial *n*, in contrast to an increasing reward prospect, increases the switch costs. This result pattern emerges for the reward remain condition compared with the reward increase condition, since the repetition trial RTs decrease while switch trial RTs increase. These findings are consistent with the assumption that the constant reward prospect leads to a stabilization of the task representation, resulting in maximized processing speed, while cognitive flexibility is reduced. Taken together, the authors suggest that the prospect of reward can activate a stable as well as a flexible mode of cognitive control, leading to the regulation of information processing policies.

Related to the current case, it is an open issue how DT processing is modulated by the reward history classification (constant vs. increasing reward prospect) as applied by Fröber and Dreisbach (2016; see also Shen & Chun, 2011). To investigate this, we classified trials as either a reward remain trial or a reward increase trial based on whether the reward prospect remained constant or increased from trial *n*−1 to trial *n*.

The results showed that RT1 was faster in the reward remain condition compared with the reward increase condition (see statistical details here^4^). This result pattern indicates that the constant reward prospect leads to a similar effect of reward-related optimization on DT and task-switching performance (i.e., maximizing processing speed). Related to the flexibility-stability framework, this result pattern could reflect an increased stability of the task representation (i.e., goal maintenance) induced by the constant reward prospect *and* the instruction to mobilize mental effort. Future studies should investigate whether the prospect of reward can also improve cognitive flexibility during DT situations. In this context, it could be worthwhile to investigate whether the prospect of reward can improve cognitive flexibility for the coordination of two tasks as required in DT situations with a variable task order (Schubert, 2008).

### Conclusion

We provided evidence for the assumption that participants can flexibly utilize a trial-wise reward prospect for their Task 1 performance resulting in rapid improvement and stabilization of DT performance. Furthermore, we obtained evidence that well-prepared DT processing was further improved by reward utilization with an increasing CTI, favoring the assumption that the prospect of reward elicited additional processing improvements going beyond the preparation-related processing improvements. Taken together, we provided novel evidence on the temporal dynamics of reward utilization in DT situations.

## Data Availability

The data generated and/or analyzed during the current study are available from the corresponding author upon reasonable request.

## Footnotes

1 It must be noted that the time for cue discrimination and for deciding on effort mobilization in the reward condition needs also to be considered when assessing the time available for reward utilization under the conditions of short and long CTI. Therefore, the resulting time available for reward utilization is even shorter than the nominal time of 200 ms and 700 ms in the two CTI conditions. We thank an anonymous reviewer for pointing this out. This needs to be also considered when specifying the hypotheses in cue-related reward tasks.

2 We applied the identical power analysis for Experiment 2. Therefore, the estimated sample size of *N* = 25 holds for Experiment 2, as well.

3 We thank an anonymous reviewer for pointing out that criteria for reward attainment might be linked to dynamic adjustments of response criteria of participants, which emerge as speed–accuracy trade-off effects, in reward compared with no-reward conditions.

4 As suggested by an anonymous reviewer, we conducted an exploratory analysis of how the reward history and CTI affect RT1. For Experiment 1, we obtained a main effect of reward history, *F*(1, 24) = 15.19, *p* < .001, η_G_^2^ = .060, with a shorter RT1 in the reward remain compared with the reward increase condition. Furthermore, we obtained a main effect of CTI, *F*(1, 24) = 17.75, *p* < .001, η_G_^2^ = .046, with a reduced RT1 in the long compared with the short CTI condition. Finally, the interaction of the factors reward history and CTI did not reach significance. For Experiment 2, we obtained a main effect of reward history, *F*(1, 23) = 13.99, *p* < .001, η_G_^2^ = .042, with a reduced RT1 in the reward remain compared with the reward increase condition. Furthermore, we obtained a main effect of CTI, *F*(1, 23) = 12.37, *p* < .002, η_G_^2^ = .011, with a shorter RT1 in the long compared with the short CTI condition. Finally, the interaction of the factors reward history and CTI, reached significance, *F*(1, 23) = 6.28, *p* < .020, η_G_^2^ = .004. The CTI effect (short minus long CTI) was increased in the reward remain (*M* = 52 ms) compared with the reward increase (*M* = 13 ms) condition. Consequently, this pattern of results indicates that a) RT1 processing profited most when the reward prospect remained constant and b) that participants required two consecutive reward-related trials to optimize their task processing, for task conditions of reduced temporal expectation.

## Code availability

Analysis scripts are available from the corresponding author on reasonable request.
